# Surgical resection with neoadjuvant chemotherapy for iliac Ewing's sarcoma in adult females: A rare case report highlighting multidisciplinary approaches and promising outcomes

**DOI:** 10.1016/j.ijscr.2024.109421

**Published:** 2024-02-21

**Authors:** A. Boushabi, H. Ait Benali, M. Shimi

**Affiliations:** Department of Orthopedics and Trauma-surgery, Mohammed VI University Hospital, Faculty of Medicine and Pharmacy, Tangier, Morocco

**Keywords:** Ewing's sarcoma, Iliac crest, Resection, Bone tumor

## Abstract

**Introduction et importance:**

The iliac origin Ewing's sarcoma is a highly malignant primitive bone tumor. Its occurrence in adults is very rare. The prognosis for this tumor in adult patients is unfavorable and has a high rate of local recurrence. The main treatment goals include preventing local recurrences and distant metastases. A secondary objective is to maintain the quality of life by avoiding major amputative surgery. The primary aim of this report is to underscore the rarity and significance of the combination of surgery with neoadjuvant chemotherapy for better outcomes.

**Case presentation:**

We present a rare case of Ewing's sarcoma of the right ilium without metastasis in a 50-year-old woman, treated with initial chemotherapy followed by extensive local marginal resection of the pelvic lesion, complemented by perimeter radiotherapy, and concluded with additional chemotherapy. The patient's progress has been satisfactory, with no recurrence observed during a 6-month follow-up period.

**Clinical discussion:**

The Ewing's sarcoma of the pelvis, being more aggressive than in other locations, presents an unfavorable prognosis, especially in cases of delayed diagnosis associated with large tumors and micrometastases. Therapeutic advancements, such as neoadjuvant chemotherapy, precise radiotherapy, and sophisticated preoperative planning, contribute to improving survival rates.

Early diagnosis of Ewing's sarcoma of the ilium remains a challenge due to subtle changes difficult to detect on radiographs and nonspecific clinical symptoms. Ewing's sarcoma of the pelvis has an unfavorable prognosis due to the lack of a major anatomical barrier to tumor spread in this region. The treatment of this disease requires a multidisciplinary approach involving oncologists, radiation oncologists, surgeons, and radiologists. The effectiveness of surgery depends on the possibility of complete resection without excessive morbidity. The clarity of surgical margins influences the prognosis, although the presence of distant metastases remains the worst prognostic factor, with a limited long-term survival of 20 % despite aggressive treatment. Histological grades do not determine the prognosis, and long-term survival is generally reported between 60 % and 70 %, emphasizing the crucial importance of early detection and appropriate therapeutic intervention.

**Conclusion:**

In summary, the case of Ewing's sarcoma in the right ilium highlights the rarity and challenges associated with this highly malignant bone tumor. Despite the unfavorable prognosis often linked to delayed diagnosis in the pelvic region, a multidisciplinary approach, including surgical resection along with neoadjuvant chemotherapy and precise radiotherapy, shows promise in improving outcomes. The satisfactory progress of the patient over 6 months underscores the potential effectiveness of this treatment plan.

## Highlights

• **Uncommon Adult Presentation:** This case underscores the rarity of Ewing's sarcoma originating from the iliac crest in a 50-year-old woman, emphasizing the unique challenges posed by this malignancy in adults.
• **Holistic Treatment Approach:** Successful management involved a comprehensive strategy, integrating neoadjuvant chemotherapy, surgical resection, and targeted radiotherapy, highlighting the significance of a multidisciplinary approach in addressing pelvic Ewing's sarcoma.
• **Diagnostic Complexity:** Early detection remains elusive due to subtle symptoms and nonspecific radiographic changes, emphasizing the critical need for precise diagnostics and collaborative efforts among medical specialties to overcome diagnostic challenges.
• **Advancements in Therapy:** The case showcases evolving therapeutic options, including limb-sparing surgeries and neoadjuvant chemotherapy, contributing to improved outcomes and raising hope for better quality of life for patients with aggressive pelvic bone tumors.
• **Positive Progress:** Despite the typically unfavorable prognosis associated with pelvic Ewing's sarcoma, the patient's encouraging progress over a 6-month follow-up period highlights the potential efficacy of the selected treatment plan, offering optimism for enhanced clinical outcomes.

## 1 Introduction

Ewing sarcoma, characterized by its high malignancy, is a rare primary bone tumor, often localized in the pelvis and lower limbs [1]. Clinically, pain followed by swelling is the predominant symptom, but the initial diagnosis can be challenging due to subtle symptoms and inconspicuous radiographic changes [2]. The average time between symptom onset and diagnosis is approximately eight months [[2], [3]]. First described by James Ewing in 1921, Ewing sarcoma is the second most common primary malignant bone tumor after osteosarcoma, typically affecting individuals between 5 and 15 years old [3].

Therapeutic approaches for Ewing sarcoma include local treatment (surgery and radiotherapy) and systemic therapy (adjuvant/neoadjuvant chemotherapy) [4]. Managing Ewing sarcoma in the pelvic region presents specific challenges due to anatomical complexity, influencing choices for resection and reconstruction. Therapeutic advancements since the 1960s, notably chemotherapy, have garnered increased interest in oncology. The use of neoadjuvant chemotherapy, followed by secondary local control for the primary site through surgical resection, radiotherapy, or both, is an accepted therapeutic scheme for patients with Ewing sarcoma [5]. Although Ewing sarcoma is considered radiosensitive, the frequent presence of pulmonary metastases can sometimes reduce treatment effectiveness. Pelvic Ewing sarcoma represents about a quarter of all primary tumors, with less favorable prognoses compared to extremity locations [6].

As Ewing sarcoma has a gender predilection of male:female of 3:1, mentioning this gender predilection in the introduction will further highlight the magnitude of the rarity of the case.

Surgical considerations for pelvic sarcoma emphasize the crucial importance of obtaining clear resection margins, particularly significant with high-grade tumors. The potential removal of the iliac crest carries major functional implications, affecting muscle insertion and hip abductors. Disturbances to the pelvic belt can lead to leg length imbalances and increased pressure on the lumbar spine and contralateral sacroiliac joint [7].

Despite advances in multimodal treatments, pelvic Ewing sarcoma retains an unfavorable prognosis, underscoring the importance of a careful and multidisciplinary approach. Here, we present a case of Ewing sarcoma in a 50-year-old woman, occurring at the right iliac crest, highlighting therapeutic challenges and considerations specific to this location. Additionally, an in-depth literature analysis has been conducted to raise awareness among practitioners regarding the clinical and histopathological variety of this unusual tumor.

This work has been reported in line with the SCARE 2023 criteria.

## 2 Case presentation

This concerns a 50-year-old woman with no notable medical history who presented to our department due to persistent pain in her right ilium for the past six months, accompanied by claudication during walking. During the interview, she reported no history of trauma, fever, cachexia, or general malaise. The systemic examination was normal. The local examination revealed an antalgic gait, tenderness over the right iliac crest with a sensation of fullness in the iliac fossa. No deformation or tenderness was observed at the hip joint, and the range of motion was similar on both sides, with no difference in limb length. The overlying skin appeared normal, and routine blood tests were within normal limits.

**Fig. 1a:**
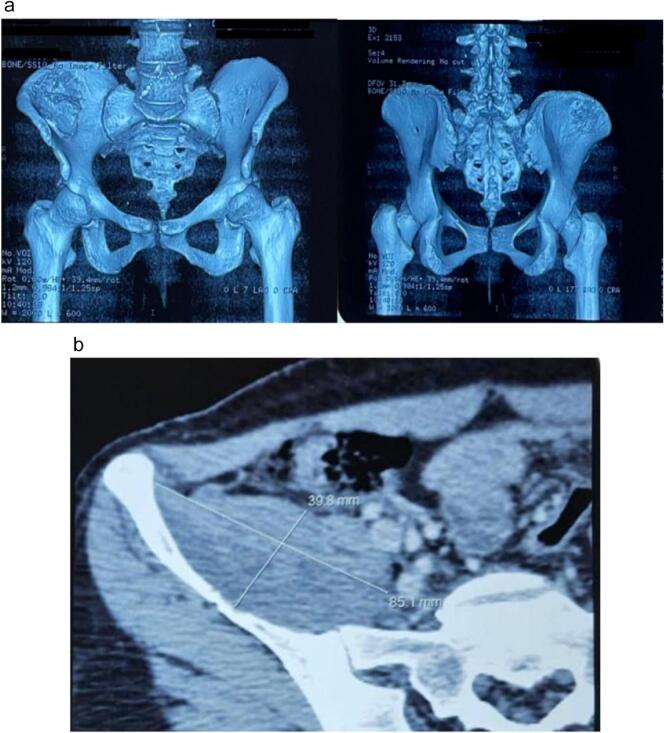
Three-dimensional CT scan of the lesion **b:** CT scan image showing the size of the tumor.

An anteroposterior pelvic X-ray revealed a blurry lytic lesion in the right iliac bone, without affecting the acetabulum or sacroiliac joint. A computed tomography (CT) scan (Fig. 1a, Fig. 1b) subsequently confirmed the presence of a heterogeneous circumferential osteolytic mass in the right iliac wing, localized to a single bone segment. This mass exhibited central necrosis with permeative cortical lysis and a spiculated periosteal reaction resembling a grass-fire pattern. It also showed extraosseous extension towards the right iliopsoas muscle internally and the external muscles, measuring approximately: 85 * 65 * 50 mm.

**Fig. 2:**
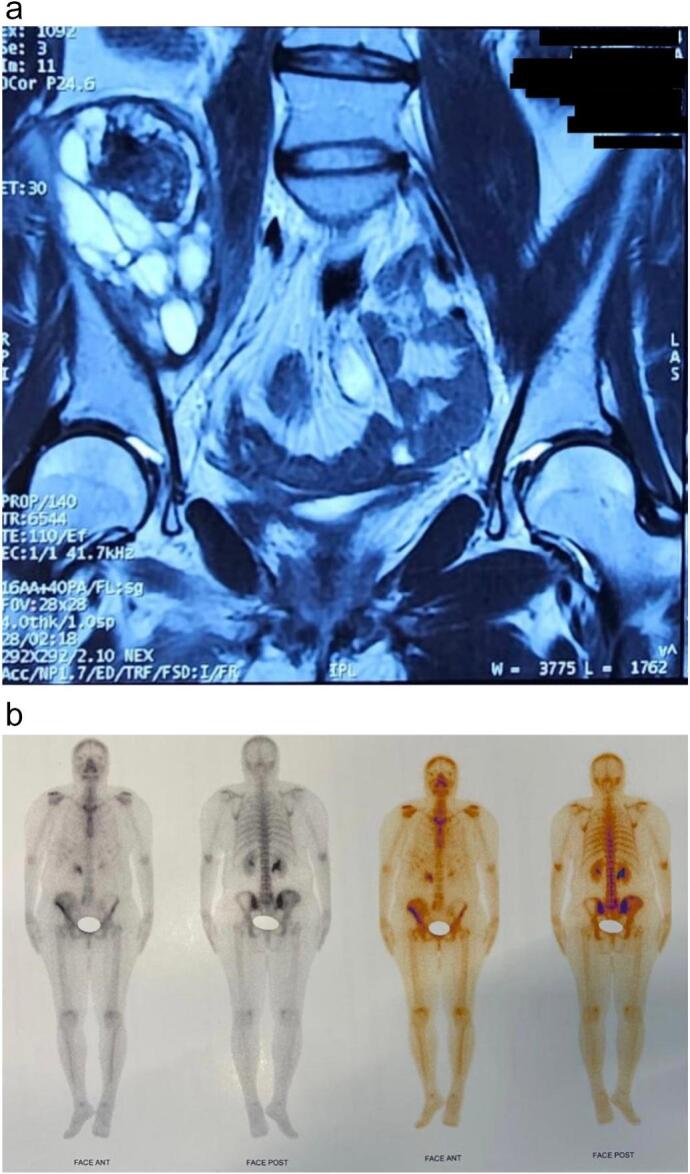
**a**: MRI images of the patient prior to the onset of neoadjuvant chemotherapy, showing a mass along the right iliac bone, most likely representing a soft tissue sarcoma with focal bone involvement. **b**: Scintigraphy images of the patient.

Magnetic resonance imaging (MRI) (Fig. 2a) revealed medullary edema with expansion of the right iliac bone, showing hypointensity in T1 and hyperintensity in T2. Soft tissues adjacent to the medial aspect of the bone were observed, suggesting the possibility of a primary malignant bone tumor. Bone scintigraphy indicated increased uptake in the pelvic region, indicating bone infiltration of the right hemipelvis, including the iliac wing, roof, and floor of the acetabulum on the right. No anomalies suggesting distant metastatic spread were detected (Fig. 2b).

In a sterile environment, a fine-needle aspiration of the mass in the right iliac fossa was performed under ultrasound guidance using a 22-gauge needle, yielding approximately 20 mL of hemorrhagic fluid containing malignant cells. An open biopsy of the mass was then performed, and the histopathologists' report confirmed the diagnosis of Ewing's sarcoma.

The multidisciplinary approach was essential in managing this case. The medical team, consisting of oncologists, orthopedic surgeons, radiologists, and pathologists, collaborated to establish an accurate diagnosis and develop an optimal treatment plan. This multidisciplinary approach resulted in surgical margins free of any residual tumor and contributed to the overall success of the treatment.

**Fig. 3:**
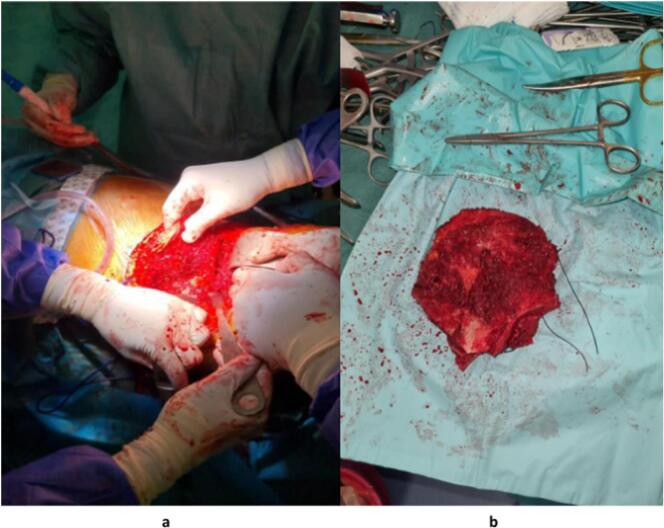
**a:** Intraoperative image of tumor resection: semilateral position 45 utilitarian pelvic approach. **b:** Intraoperative image of the surgical specimen post-resection.

**Fig. 4:**
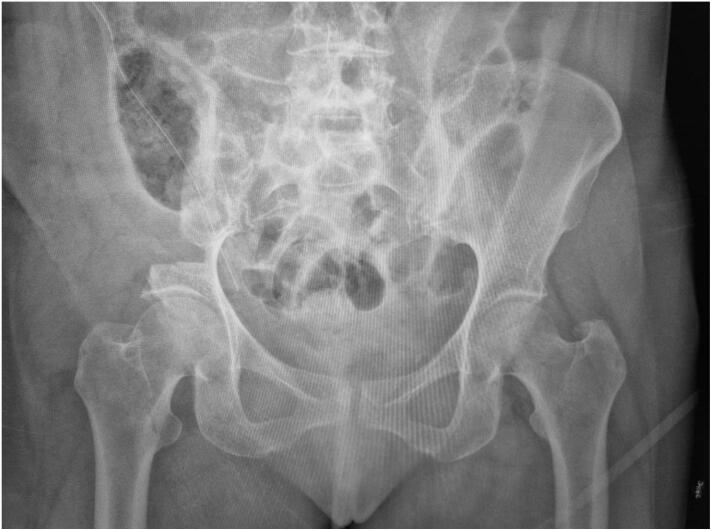
**:**X-ray image of the patient post-tumor resection after 6 months.

The patient underwent neoadjuvant chemotherapy consisting of vincristine, cyclophosphamide, doxorubicin, etoposide, and ifosfamide (VCD/IE three times a week), resulting in a reduction in the size of the mass. Subsequently, an internal hemipelvectomy (Fig. 3) was performed with a utility pelvic approach in a semilateral position at 45 °, excising the tumor mass with the affected bone while preserving the sacroiliac joint and hip joint (Fig. 3) (Fig. 3b). Margins were free of any residual tumor. The patient then received postoperative chemotherapy, followed by radiotherapy to reduce the risk of recurrence. Currently, the patient is doing well, walking normally, and experiencing no pain. Regular follow-up will be maintained for the patient (Fig. 4).

## 3 Discussion

Early diagnosis of Ewing's sarcoma of the ilium, particularly the iliac crest, remains a challenge due to its atypical presentation and possible confusion with other conditions [8]. This discussion focuses on the case of a 50-year-old patient, emphasizing diagnostic nuances, therapeutic challenges, and highlighting the central role of chemotherapy and radiopharmaceuticals in management.

Nonspecific radiological changes in the iliac bone complicate early diagnosis, underscoring the importance of precise diagnostic approaches. Our patient's case underscores the need to distinguish Ewing's sarcoma from other conditions such as sacroiliitis or septic arthritis, emphasizing clinical challenges [9]. Effective management of Ewing's sarcoma requires close collaboration among oncologists, radiologists, and surgeons. Management should encompass not only appropriate medical and surgical treatment but also psychological and social support to provide psychological assistance to patients, given the aggressive and often traumatic nature of the diagnosis, treatments, and potential impact on quality of life. This may involve counseling sessions, support groups, or other psychological services [10].

Molecular biology techniques are essential for diagnosing Ewing's tumor by identifying a fusion transcript resulting from a specific translocation t(11;22) or t(21;22) [11]. The treatment of this tumor primarily relies on surgical resection, usually combined with chemotherapy. Resections limited to a portion of the iliac wing or the obturator ring generally do not require reconstruction. However, for resections involving the cotyloid region or those affecting the entire iliac wing, reconstruction is often necessary to ensure a satisfactory functional outcome [7].

With the advent of effective chemotherapy, advanced imaging techniques, and modern surgical methods, limb-sparing procedures have replaced amputation for the radical treatment of malignant pelvic bone tumors [[4], [5], [6], [7], [8], [9], [10], [11], [12]]. Although surgery is a preferred option when possible, our case illustrates that clear margins can be achieved without causing functional impairment. However, the prognosis remains influenced by various factors such as size, location, and the presence of distant metastases [12]. Patients without metastases at the time of diagnosis or intervention had a cumulative overall survival of 82.7 % at 5 years and 80.1 % at 10 years [13]. The mortality rate for this tumor becomes extremely high when treated solely by surgery or radiotherapy. For many unresectable Ewing's sarcomas, radiotherapy is frequently applied after chemotherapy to increase the elimination of cancer cells and control their development [13].

Patients with Ewing's sarcoma benefit from a multimodal approach, where chemotherapy plays a crucial role. Advances in neoadjuvant chemotherapy protocols have contributed to improving overall survival rates [4]. In our case, chemotherapy and targeted radiotherapy were successfully administered before surgery, emphasizing their beneficial impact. Pelvic Ewing's sarcomas, being more aggressive, require an intensive therapeutic approach. Recent advances, such as neoadjuvant chemotherapy and targeted radiotherapy, have significantly improved survival rates. Despite these advances, late diagnosis remains associated with challenges in local and systemic control [14].

Retrospective data highlight the importance of resection to improve survival rates, especially in extensive pelvic lesions. However, functional complications and reconstruction challenges must be carefully evaluated, especially when the sacroiliac joint is involved [15]. Although the incorporation of surgery has improved outcomes, challenges persist, especially in pelvic lesions. Studies differ regarding the impact of resection on disease-free overall survival, emphasizing the need for ongoing research to optimize chemotherapy effectiveness and its role in preventing recurrences [16].

The management of Ewing's sarcoma of the right iliac crest in our 50-year-old patient highlights diagnostic and therapeutic challenges. A multimodal approach centered on chemotherapy offers promising perspectives to enhance clinical outcomes and the quality of life for patients with pelvic Ewing's sarcoma [17].

## 4 Conclusion

Ewing's sarcoma, an extremely aggressive malignant primitive bone tumor, poses significant challenges for early diagnosis. Its prognosis depends on factors such as size, location, stage, and the presence of distant metastases. Thanks to advances in modern chemotherapy and radiopharmaceuticals, the prognosis has significantly improved. For optimal management of Ewing's sarcoma, it is essential to ensure effective management by fostering close collaboration among oncologists, radiologists, and surgeons.

## Consent

Written informed consent was obtained from the patient for publication and any accompanying images. A copy of the written consent is available for review by the Editor-in-Chief of this journal on request.

## Ethical approval

Not applicable.

## Funding

This research did not receive any specific grant from funding agencies in the public, commercial, or not-for-profit sectors.

## Author contribution

BOUSHABI Ayoub: study concept, Data collection; data analysis; writing review & editing.

AIT BENALI Hicham: Contributor, Supervision and data validation.

SHIMI Mohammed: supervision and data validation.

## Guarantor

BOUSHABI Ayoub.

AIT BENALI Hicham.

SHIMI Mohammed.

## Declaration of competing interest

The authors state that they have no conflicts of interest for this report.
